# CACNA1C is a prognostic predictor for patients with ovarian cancer

**DOI:** 10.1186/s13048-021-00830-z

**Published:** 2021-07-01

**Authors:** Xiaohan Chang, Yunxia Dong

**Affiliations:** 1 Department of Obstetrics and Gynecology, Shengjing Hospital of China Medical University, No. 36 Sanhao street, Liaoning Province 110004 Shenyang, P.R. China; 2 Department of Anesthesiology, Shengjing Hospital of China Medical University, No. 36 Sanhao street, Liaoning Province 110004 Shenyang, P.R. China

**Keywords:** CACNA1C, Ovarian cancer, Immunity, Prognosis, Overall survival

## Abstract

**Background:**

CACNA1C, as a type of voltage-dependent calcium ion transmembrane channel, played regulatory roles in the development and progress of multiple tumors. This study was aimed to analyze the roles of CACNA1C in ovarian cancer (OC) of overall survival (OS) and to explore its relationships with immunity.

**Methods:**

Single gene mRNA sequencing data and corresponding clinical information were obtained from The Cancer Genome Atlas Database (TCGA) and the International Cancer Genome Consortium (ICGC) datasets. Gene set enrichment analysis (GSEA) was used to identify CACNA1C-related signal pathways. Univariate and multivariate Cox regression analyses were applied to evaluate independent prognostic factors. Besides, associations between CACNA1C and immunity were also explored.

**Results:**

CACNA1C had a lower expression in OC tumor tissues than in normal tissues (*P* < 0.001), with significant OS (*P* = 0.013) and a low diagnostic efficiency. We further validated the expression levels of CACNA1C in OC by means of the ICGC dataset (*P* = 0.01), qRT-PCR results (*P* < 0.001) and the HPA database. Univariate and multivariate Cox hazard regression analyses indicated that CACNA1C could be an independent risk factor of OS for OC patients (both *P* < 0.001). Five significant CACNA1C-related signaling pathways were identified by means of GSEA. As for genetic alteration analysis, altered CACNA1C groups were significantly associated with OS (*P* = 0.0169), progression-free survival (*P* = 0.0404), disease-free survival (*P* = 0.0417) and disease-specific survival (*P* = 9.280e-3), compared with unaltered groups in OC. Besides, CACNA1C was dramatically associated with microsatellite instability (MSI) and immunity.

**Conclusions:**

Our results shed light on that CACNA1C could be a prognostic predictor of OS in OC and it was closely related to immunity.

**Supplementary Information:**

The online version contains supplementary material available at 10.1186/s13048-021-00830-z.

## Introduction

Ovarian cancer (OC) is the third most common gynecologic malignancy worldwide, with approximately 21,750 newly estimated cases and 13,940 newly estimated death in USA, 2020 [1]. Despite other cancers such as endometrial cancer (EC) having higher rates of incidence, OC is the deadliest of all female reproductive cancers [2]. This disease is rare in young women, especially under the age of 30 and its risks will increase with age, sharply after the age of 50. So, the average diagnosis age of OC is between 50 and 70 [3]. Because the clinical manifestations of early OC are hidden or unspecific, OC is often called a silent killer, and approximately 70 % of them are diagnosed with an advanced stage [4]. As reported, the 5-year survival rate for FIGO stage I is 90 %, stage II is 65 %, stage III is 34 %, and stage IV is 15 % [5, 6]. However, current improvements in treatment methods could only slightly improve OC survival rates [7]. Therefore, there was an urgently need to clarify the potential molecular mechanisms of OC and identify novel biomarkers for early diagnosis and prognosis assessment [8, 9].

Calcium channels could be generally classified into two categories containing the voltage-gated calcium channels (VGCCs) and the ligand-gated calcium channels (LGCCs) [10]. As a type of VGCCs, calcium voltage-gated channel subunit alpha1C (CACNA1C) was coded by the α1 subunit Cav1.2 and it was reported to be involved in regulating cell-matrix adhesion, collagen fibril organization, cell adhesion, cellular response to amino acid stimulus, and negative regulation of cell proliferation [11–13]. Previous meta-analysis results showed that CACNA1C was up-regulated in brain tumors, leukemia, breast cancer and other tumors, suggesting its regulatory roles in cancer progression [14]. Bioinformatics analysis also revealed that mutation of CACNA1C was significantly associated with longer overall survival (OS) in EC patients [15]. All of these indicated that CACNA1C could be served as a prognostic biomarker for human cancers. Therefore, this study was aimed to explore the prognostic values of CACNA1C in OC, to identify CACNA1C-related signal pathways and to further explore its associations with immunity, thus helping to provide some references for future basic CACNA1C-related researches in OC.

## Materials and methods

### Data acquisition and processing

CACNA1C single gene expression matrix and corresponding clinical data of 379 OC tumor samples were obtained from The Cancer Genome Atlas (TCGA) database (http://cancergenome.nih.gov/), while 88 ovary normal tissue samples were getting from the Genetype-Tissue Expression (GTEx) data portal (https://gtexportal.org/). In addition, another OC dataset from the International Cancer Genome Consortium (ICGC) data portal (https://icgc.org/) was used as external verification database, involving 111 tumor samples. All data were preprocessed by normalization or log2 transformation and analyzed by R software (https://www.rproject.org/) [16]. The “limma” package was used to calculate differentially expressed genes (DEGs), with |log2 fold change (FC)| >1 and adjusted P-values (FDR) < 0.05 as the cut-off criteria.

### Quantitative real-time PCR (qRT‑PCR)

The qRT‑PCR analysis was performed to detect the expression levels of CACNA1C in OC tissues, according to the manufacturer’s instructions (TAKARA) [17]. All reactions were carried out in triplicate and Actin was used as the internal controls for mRNA. The relative gene expression in tissues was calculated using the comparative delta-delta CT (2-ΔΔCt) method. Primers were synthesized by TSINGKE (Beijing, China), including Actin (F:5’ ATGACTTAGTTGCGTTACACC 3’, R:5’ GACTTCCTGTAACAACGCATC 3’) and CACNA1C (F:5’ GAAGCGGCAGCAATATGGGA 3’, R:5’ TTGGTGGCGTTGGAATCATCT 3’). Six paired tumor and adjacent normal tissues were obtained from OC patients from Shengjing Hospital of China Medical University. Ethical approval was obtained from the Institutional Research Ethics Committees of Shengjing Hospital of China Medical University and informed written consent was obtained from all subjects.

### The Human Protein Atlas (HPA) database and protein-protein interaction (PPI) network analysis

The Human Protein Atlas (HPA, http://www.proteinatlas.org/) online database was utilized to validate the protein expression of CACNA1C in OC by immunohistochemical staining in this study. With the help of online STRING (https://string-db.org/) database, PPI full network was conducted to explore the potential relationships between CACNA1C and other related genes in OC, with the medium confidence > 0.40.

### The receiver operating characteristic (ROC) curves and logistic regression analysis

By utilizing the R “survivalROC” package, ROC curves and the area under the curve (AUC) values were performed to assess the specificity and sensitivity of CACNA1C for OC. Moreover, logistic regression analysis was utilized to explore the associations between CACNA1C expression and 10 clinicopathological parameters (stage, grade, age, location, race, status, diagnosis, venous-invasion, chemotherapy, outcome) in OC patients and *P*-values below 0.05 were regarded as statistical significance [18].

### Univariate and multivariate Cox hazard regression analysis

We performed univariate and multivariate Cox hazard regression analysis to determine whether or not CACNA1C and 10 clinicopathological parameters (stage, grade, age, location, race, status, diagnosis, venous-invasion, chemotherapy, outcome) could be independent factors related to OS on OC patients in the TCGA dataset by means of R package.

### Gene set enrichment analysis (GSEA) analysis

GSEA, as a computational method determining whether or not a priori defined set of genes shows statistically significant, concordant differences between two biological states, was carried out to explore the significant survival differences between high- and low-CACNA1C groups [19]. The expression level of CACNA1C was used as a phenotype label and gene set permutations were performed 1000 times for each analysis with the consideration of the nominal p value < 0.05 and normalized enrichment score (NES) > 1.5 as the threshold.

### Genetic alteration analysis

We utilized the cBioPortal for Cancer Genomics (http://cbioportal.org) to explore the CACNA1C alteration frequency, mutation type and CNA (copy number alteration) across all TCGA tumors by means of the “Cancer Types Summary” module. Furthermore, the overall survival, progression-free survival, disease-free survival, and disease-specific survival differences for OC with or without CACNA1C genetic alteration were also showed by Kaplan-Meier (K-M) survival plots and log-rank *p*-values [20, 21].

### The evaluation of microsatellite instability (MSI), tumor mutational burden (TMB), tumor neoantigen burden (TNB) and immunity

In order to explore the associations between CACNA1C gene expression and MSI or TMB or TNB, correlation analyses was performed by the Spearman’s method and visualized by radar maps [22, 23]. As previously described, we further explored the relationships between CACNA1C gene expression and immunity. Four aspects were analyzed including tumor immune infiltration, tumor microenvironment, immune checkpoint molecules and immune cells pathway by R packages“estimate”, “limma”, “reshape2” and “RColorBrewer” [24, 25]. All of these above mentioned analyses were performed by using the Sangerbox tool, which is a free online data analysis platform (http://www.sangerbox.com/tool).

## Results

### The expression level of CACNA1C in OC based on TCGA and GTEx datasets

**Fig. 1:**
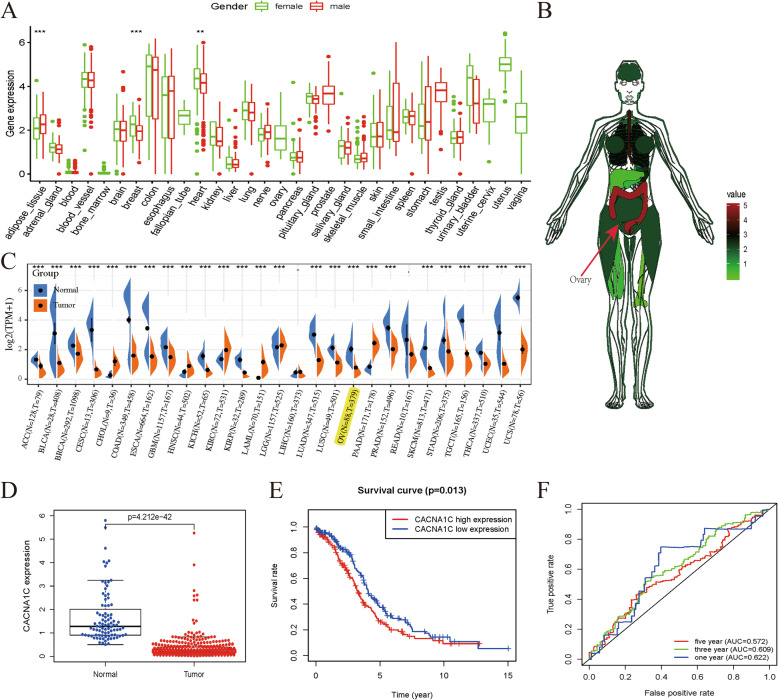
The expression level of CACNA1C in OC based on TCGA and GTEx datasets; (**A**) CACNA1C expression levels in various normal tissues and organs by GTEx database; (**B**) Anatomical heatmap of CACNA1C expression in female by GTEx database; (**C**) CACNA1C mRNA expression levels in pan-cancers from TCGA and GTEx datasets; (**D**) Boxplot of CACNA1C expression between the OC tumor and normal tissues from TCGA and GTEx datasets (Normal = 88 and Tumor = 379); (**E**) K-M survival analysis of CACNA1C in TCGA; (**F**) ROC curves and its AUCs for 1-, 3-, and 5-year survival of CACNA1C; **P < 0.01; ***P < 0.001

As showed in Fig. 1 A, we obtained the expression level of CACNA1C in various normal tissues and organs by GTEx database and noticed that there were significant differences between female and male in adipose tissues, breast tissues, and heart tissues (all *P* < 0.01). Anatomical heatmap of CACNA1C expression in female indicated that it was lowly expressed in ovary (Fig. 1B). We combined TCGA and GTEx datasets to investigate the different expression levels of CACNA1C between pan-cancers and adjacent normal tissues (Fig. 1 C). As displayed in Fig. 1D, the expression level of CACNA1C in OC tissues was obviously lower than that in normal tissues (*P* < 0.001). Based on the median expression as the cut-off value, OC patients were classified into low- and high-CACNA1C groups and significant expression differences were displayed in these two groups (*P* < 0.0001). K-M survival analysis presented that high-CACNA1C groups had a shorted OS than those in the low-CACNA1C groups (*P* = 0.013; Fig. 1E). ROC curves were carried out to demonstrate the performance of the CACNA1C expression level for survival prediction and its AUC values for 1-, 3-, and 5-year survival were 0.622, 0.609, 0.572, merely having a low diagnostic efficiency (Fig. 1 F).

### Validation of CACNA1C expression in OC

**Fig. 2:**
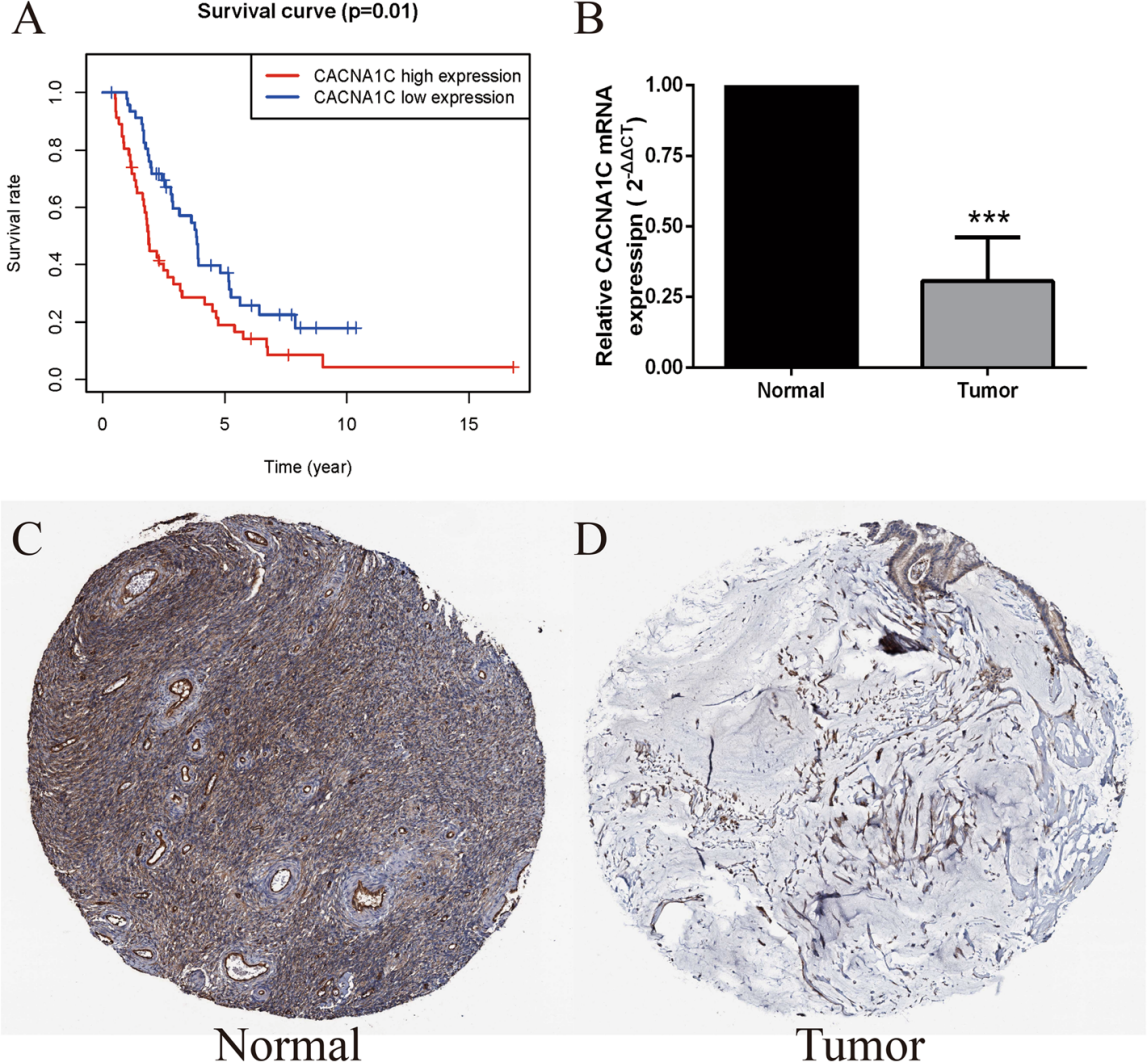
Validation of CACNA1C expression in OC; (**A**) K-M survival analysis of CACNA1C in OC from ICGC dataset; (**B**) qRT-PCR verification of CACNA1C expression in normal and OC tissues (Normal = 6; Tumor = 6); (**C-D**) Immunohistochemical staining from the HPA database for CACNA1C in OC; ***P < 0.001

We utilized the ICGC data portal (https://icgc.org/) as external verification database. Based on the median expression, OC patients in ICGC dataset were classified into low- and high-risk subgroups and high-CACNA1C groups also had a lower OS than those in the low-CACNA1C groups (*P* = 0.01, Fig. 2 A). Moreover, qRT-PCR results verified that the expression levels of CACNA1C were significantly down-regulated in the OC tissues compared with normal tissues (Normal = 6; Tumor = 6; *P* < 0.001; Fig. 2B). Besides, immunohistochemical pictures from the HPA database (https://www.proteinatlas.org/) showed that CACNA1C utilizing HPA039796 antibody had a medium expression in 3/3 normal ovary tissues, while its expression was not detected in 10/12, low in 1/12, medium in 1/12 OC tumor tissues. Obviously, CACNA1C was also lowly expressed in OC tumor tissues compared with normal tissues (Fig. 2 C-D).

### Associations between CACNA1C expression and clinicopathologic characteristics in OC

**Fig. 3:**
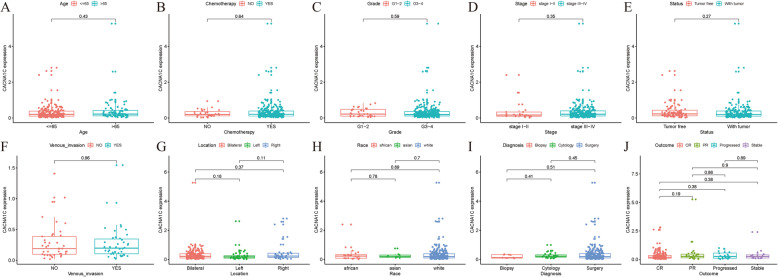
Associations between CACNA1C expression and clinicopathologic characteristics; (**A**) Age; (**B**) Chemotherapy; (**C**) Grade; (**D**) Stage; (**E**) Status; (**F**) Venous-invasion; (**G**) Location; (**H**) Race; (**I**) Diagnosis; (**J**) Outcome

Logistic regression analysis results indicated that *P* value(s) for the associations between CACNA1C expression and age was 0.43; chemotherapy was 0.64; grade was 0.59; stage was 0.35; status was 0.27; venous-invasion was 0.96; location were all above 0.05; race were all above 0.05; diagnosis were all above 0.05; outcome were all above 0.05 **(**Fig. 3). Obviously, all *P* values were above 0.05. In other words, there were no significant associations between the CACNA1C expression and stage, grade, age, location, race, status, diagnosis, venous-invasion, chemotherapy or outcome in TCGA OC samples.

### CACNA1C could serve as an independent prognostic factor

**Fig. 4:**
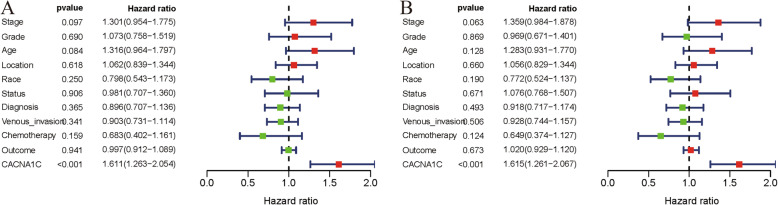
CACNA1C could serve as an independent prognostic factor of OS in ccRCC; (**A**) Univariate Cox hazard regression analysis of CACNA1C and clinicopathologic variables of ccRCC in TCGA cohort; (**B**) Multivariate Cox hazard regression analysis of CACNA1C and clinicopathologic variables of ccRCC in TCGA cohort

To identify independent prognostic factors, univariate and multivariate Cox hazard regression analyses were utilized to exclude CACNA1C and 10 clinicopathological parameters (stage, grade, age, location, race, status, diagnosis, venous-invasion, chemotherapy, outcome) with little OS values. According to our results, the CACNA1C expression was significantly associated with OS in both univariate and multivariate Cox hazard regression analyses (both *P* < 0.001), indicating that CACNA1C could be an independent risk factor of OS for OC prognosis (Fig. 4; Table 1).

**Table 1:** Univariate and multivariate cox analysis of CACNA1C and clinicopathologic characteristics of overall survival in TCGA OV cohort

Clinical characteristics	Univariate analysis		Multivariate analysis	
	HR (95 % CI)	*p*-Value	HR (95 % CI)	*p*-Value
Stage	1.301(0.954–1.775)	0.097	1.359(0.984–1.878)	0.063
Grade	1.073(0.758–1.519)	0.690	0.969(0.671–1.401)	0.869
Age	1.316(0.964–1.797)	0.084	1.283(0.931–1.770)	0.128
Location	1.062(0.839–1.344)	0.618	1.056(0.829–1.344)	0.660
Race	0.798(0.543–1.173)	0.250	0.772(0.524–1.137)	0.190
Status	0.981(0.707–1.360)	0.906	1.076(0.768–1.507)	0.671
Diagnosis	0.896(0.707–1.136)	0.365	0.918(0.717–1.174)	0.493
Venous_invasion	0.903(0.731–1.114)	0.341	0.928(0.744–1.157)	0.506
Chemotherapy	0.683(0.402–1.616)	0.159	0.649(0.374–1.127)	0.124
Outcome	0.997(0.912–1.089)	0.941	1.020(0.929–1.120)	0.673
CACNA1C	1.611(1.263–2.054)	< 0.001	1.615(1.261–2.067)	< 0.001

### GSEA identified CACNA1C-related signaling pathways

**Fig. 5:**
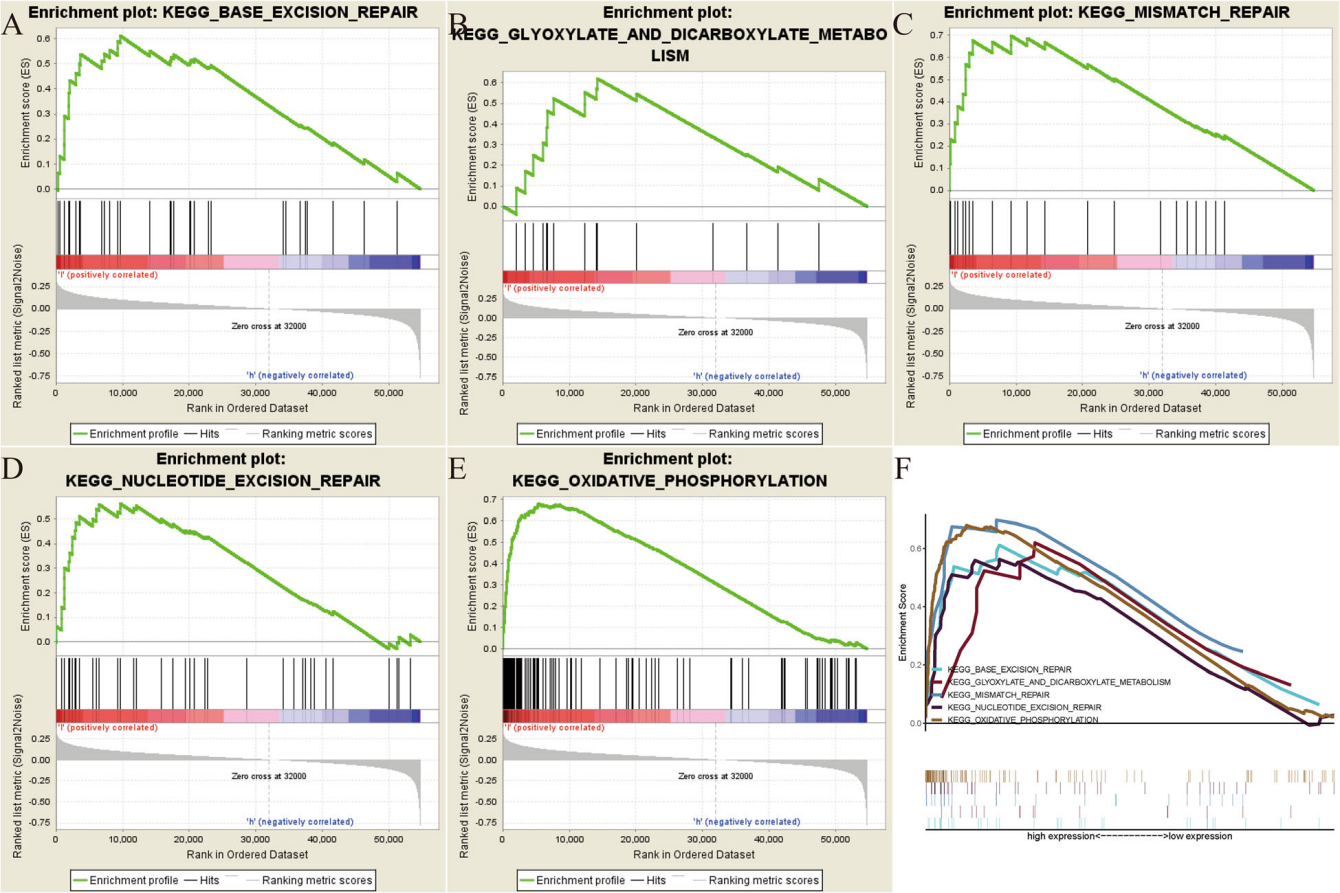
GSEA identified CACNA1C-related signaling pathways; (**A**) Base excision repair signaling pathway; (**B**) Glyoxylate and dicarboxylate metabolism signaling pathway; (**C**) Mismatch repair signaling pathway; (**D**) Nucleotide excision repair signaling pathway; (**E**) Oxidative phosphorylation signaling pathway; (**F**) All of the five significant signaling pathways

In order to explore how CACNA1C participates in the pathogenesis of OC, GSEA analysis was conducted to select related signaling pathways. GSEA analysis was performed between the high- and low-CACNA1C expression datasets, with the consideration of the nominal *p* value < 0.05 and NES > 1.5 as the threshold. Finally, five pathways that exhibited significant differential enrichment in the high-CACNA1C expression phenotype were identified, containing Base excision repair signaling pathway, Glyoxylate and dicarboxylate metabolism signaling pathway, Mismatch repair signaling pathway, Nucleotide excision repair signaling pathway and Oxidative phosphorylation signaling pathway (Fig. 5; Table 2), thus helping to understand the pathogenesis of OC.

**Table 2:** Gene sets enriched in phenotype high

MSigDB collection	Gene set name	NES	NOM p-val	FDR q-val
c2.cp.kegg.v7.1.symbols.gmt	BASE_EXCISION_REPAIR	1.974	0.000	0.011
	GLYOXYLATE_AND_DICARBOXYLATE_METABOLISM	1.809	0.002	0.032
	MISMATCH_REPAIR	1.888	0.002	0.021
	NUCLEOTIDE_EXCISION_REPAIR	1.893	0.006	0.023
	OXIDATIVE_PHOSPHORYLATION	2.189	0.000	0.001

### Genetic alteration analysis of CACNA1C in OC by cBioPortal database

**Fig. 6:**
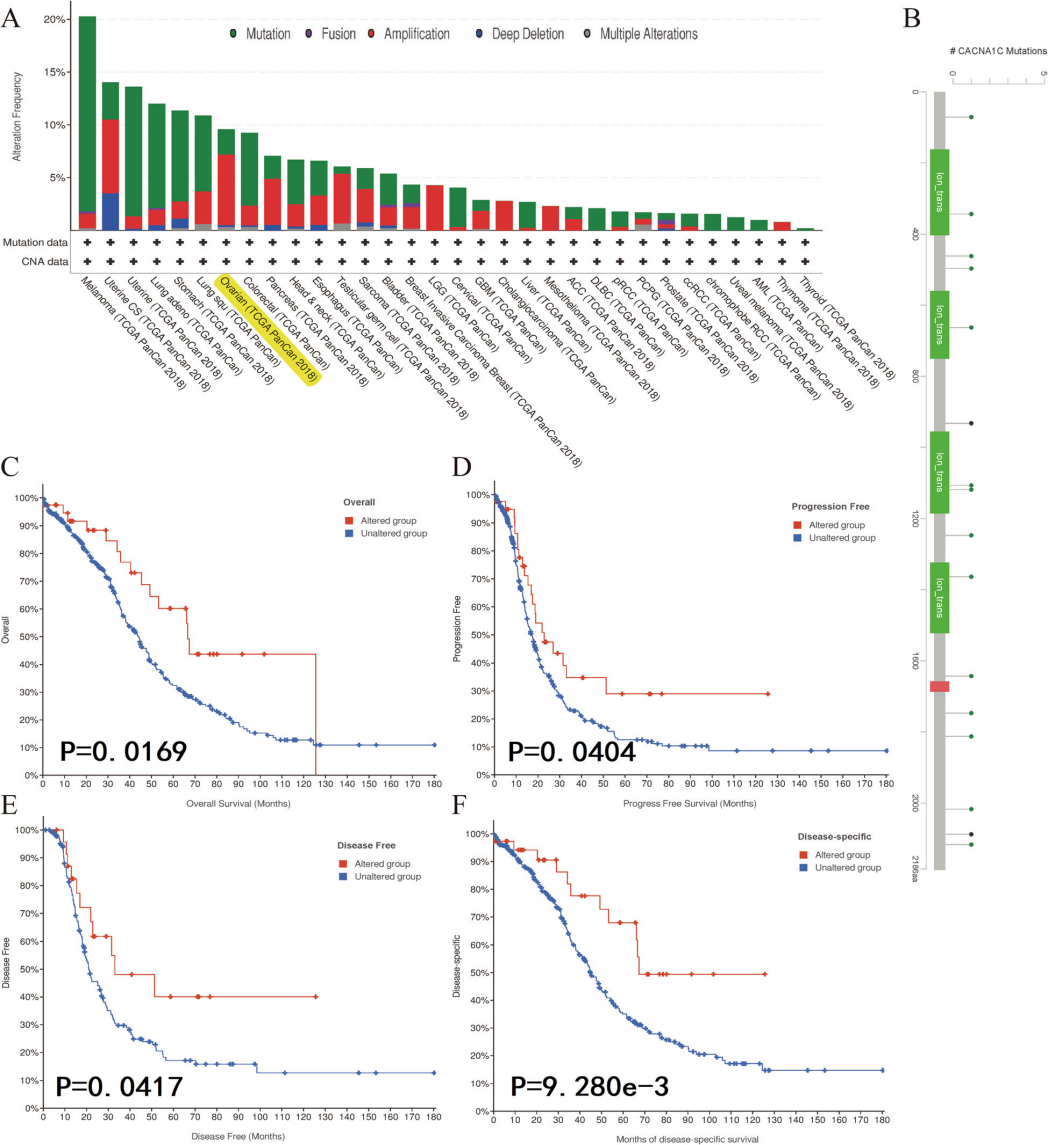
Genetic alteration analysis of CACNA1C in OC by cBioPortal database; (**A**) The alteration frequency with mutation type of CACNA1C in different tumor samples from TCGA cohorts; (**B**) CACNA1C mutation sites in OC from TCGA cohort; (**C**) K-M survival analysis of OS with CACNA1C altered or unaltered groups; (**D**) K-M survival analysis of progression-free survival with CACNA1C altered or unaltered groups; (**E**) K-M survival analysis of disease-free survival with CACNA1C altered or unaltered groups; (**F**) K-M survival analysis of disease-specific survival with CACNA1C altered or unaltered groups

As displayed in Fig. 6 A, we noticed that the genetic alteration frequency of CACNA1C was about 10 % in OC by means of the cBioPortal tool from TCGA dataset. Moreover, CACNA1C mutation sites in OC were also detailed in Fig. 6B. Further K-M survival analyses indicated that altered CACNA1C groups were significantly associated with OS (*P* = 0.0169), progression-free survival (*P* = 0.0404), disease-free survival (*P* = 0.0417) and disease-specific survival (*P* = 9.280e-3), compared with unaltered groups in OC (Fig. 6 C-F). Besides, altered CACNA1C-related signaling pathways were also identified in Figure S1, including Cell-cycle-signaling-pathway, RTK-RAS-PI(3)K-pathway and Notch-signaling-pathway. All of these indicated that genetic alteration of CACNA1C might play vital roles in OC.

### Associations between the CACNA1C expression and PPI, MSI, TMB, TNB in OC

**Fig. 7:**
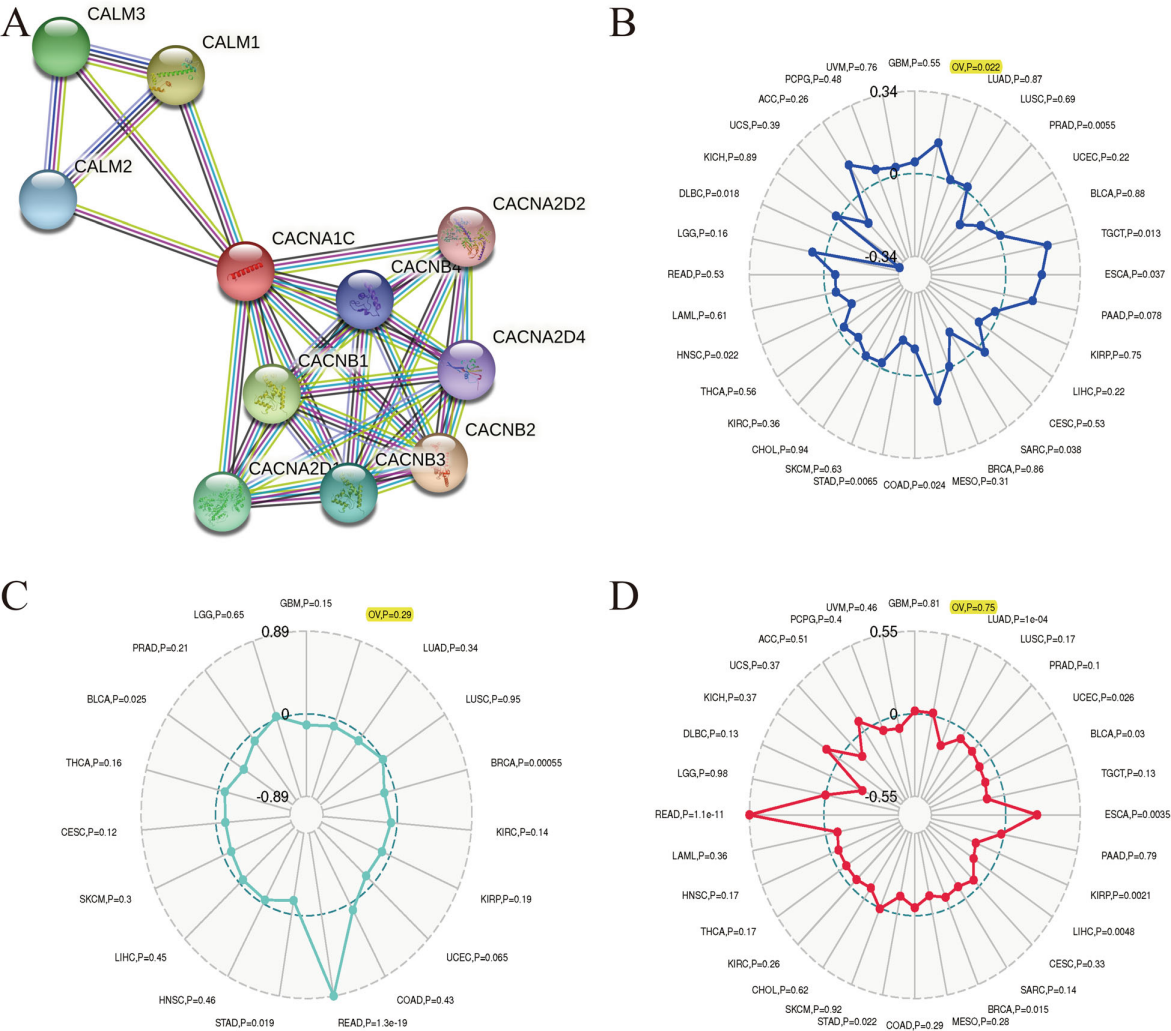
Associations between the CACNA1C expression and PPI, MSI, TMB, TNB in OC; (**A**) PPI network; (**B**) Associations between the CACNA1C expression and MSI; (**C**) Associations between the CACNA1C expression and TNB; (**D**) Associations between the CACNA1C expression and TMB

PPI network indicated that ten genes (CALM1、CALM2、CALM3、CACNA2D1、CACNA2D2、CACNA2D4、CACNB1、CACNB2、CACNB3 and CACNB4) were significantly associated with the CACNA1C expression (Fig. 7 A). Based on OC samples from the TCGA cohort, we explored the associations between the CACNA1C expression and MSI, TMB, TNB. Our results shed light on that the CACNA1C expression was significantly related to MSI (*P* = 0.022) in OC, while it had nothing to do with TMB (*P* = 0.75) or TNB (*P* = 0.29) (Fig. 7B and D).

### Associations between CACNA1C and tumor immune infiltrations, tumor microenvironment, immune checkpoint molecules, immune cells pathway in OC

**Fig. 8:**
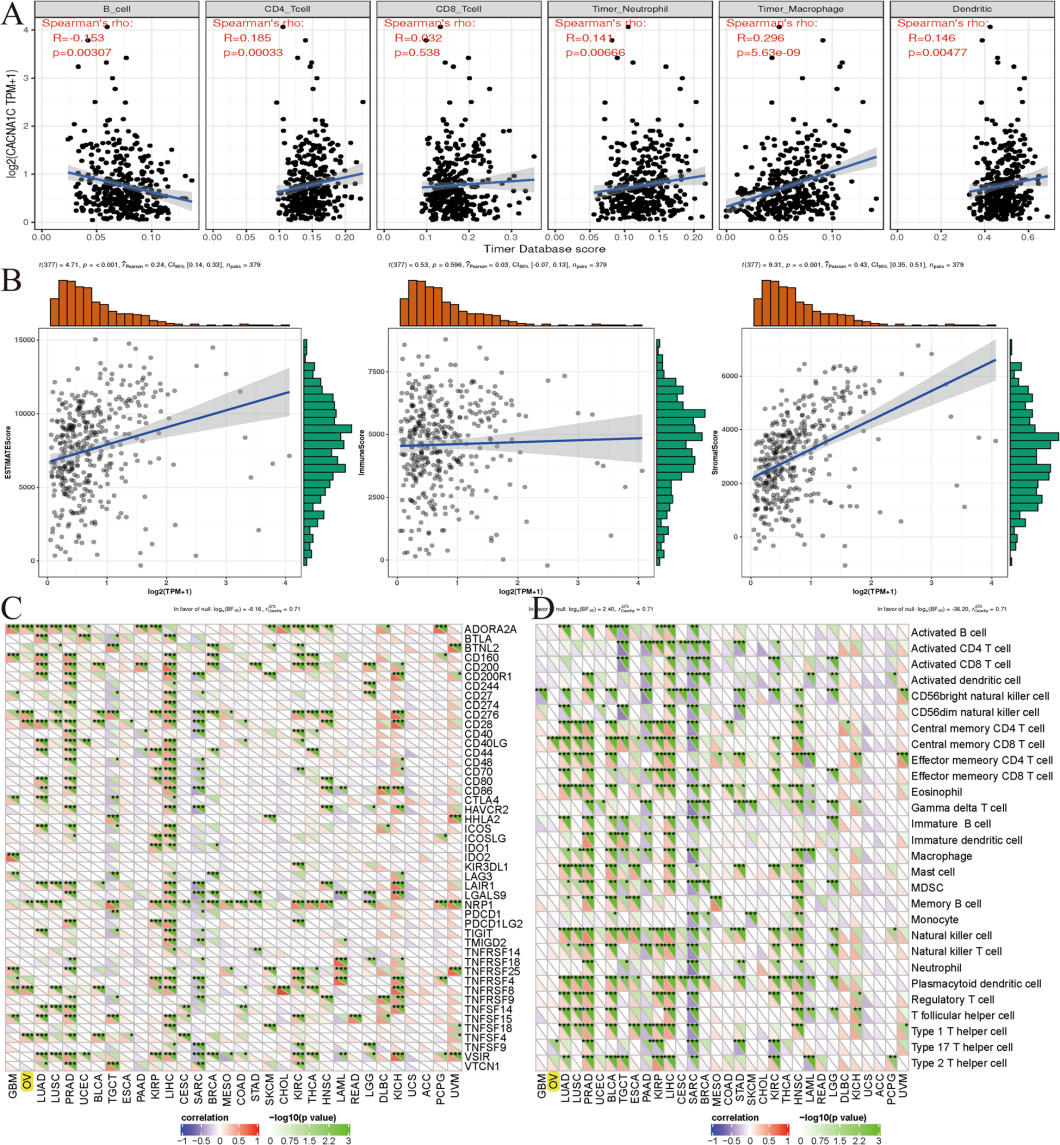
Associations between CACNA1C and tumor immune infiltrations, tumor microenvironment, immune checkpoint molecules, immune cells pathway in OC; (**A**) Associations between the CACNA1C expression and tumor immune infiltration; (**B**) Associations between the CACNA1C expression and immune microenvironment; (**C**) Correlations between the CACNA1C expression and immune checkpoint molecules; (**D**) Correlations between the CACNA1C expression and immune cells pathway

To further explore the relationships between the CACNA1C expression and immunity, four aspects were analyzed respectively, including tumor immune infiltration, tumor microenvironment, immune checkpoint molecules and immune cells pathway. We assessed the correlations between CACNA1C and the infiltration levels of six immune cells through online analysis of TIMER, and found that the CACNA1C expression was significantly associated with B cell infiltration, CD4 + T cell infiltration, neutrophil infiltration, macrophage infiltration, and dendritic cell infiltration (all *P* < 0.0; Fig. 8 A). As for tumor microenvironment, the CACNA1C expression was markedly related to ESTIMATEScore and StromalScore (both *P* < 0.001; Fig. 8B). Correlations between the CACNA1C expression and immune checkpoint molecules or immune cells pathway indicated that this gene was remarkably linked to ADORA2A, CD276, CD28, NRP1, TNFRSF8, TNFSF4, Central memory CD8 T cells pathway and Type 17 T helper cells pathway (Fig. 8 C-D).

## Discussion

As a common gynecological cancer, OC was a highly malignant disease with a high recurrence rate and a poor 5-year survival rate [26]. Although various treatments such as chemotherapy, radiotherapy, immunotherapy, and targeted therapy had been applied, the 5-year survival rate of advanced OC patients was still less than 30 % [27]. At present, it was difficult to diagnose early and there were also no accurate strategies to predict the prognosis and recurrence of OC [28]. Moreover, the classical TNM staging system, relying mainly on anatomical information without any molecular biological features, had difficulties in accurately stratifying indolent and aggressive cancers to a certain extent [29, 30]. Therefore, it was urgent to identify novel and effective biomarkers for OC prognosis and to further explore its related mechanisms [31].

In this study, we explored the prognostic values of CACNA1C in OC, identified CACNA1C-related signal pathways and further explored its associations with immunity. Our results indicated that CACNA1C had a lower expression in OC tumor tissues than in normal tissues, with significant OS and a low diagnostic efficiency. We further validated the expression levels of CACNA1C in OC by means of the ICGC dataset, qRT-PCR results and the HPA database. Furthermore, univariate and multivariate Cox hazard regression analyses indicated that CACNA1C could be an independent risk factor of OS affecting the prognosis of OC patients. In line with previously published articles, CACNA1C was up-regulated in brain tumors, leukemia, breast cancer and other tumors [14]. Besides, variants of CACNA1C were associated with bladder cancer, endometrial cancer and breast cancer risk [15, 32, 33]. All of these indicated that CACNA1C could also be a prognostic predictor for patients with OC.

GSEA, as a useful tool, had been widely applied in OC to identify related signaling pathways [34, 35]. Shi et al. found that knocking down CerS6 could significantly affect cell cycle in serous OC cells by GSEA and RNA sequencing analyses [36]. Zhang et al. performed a GSEA of SUCNR1 based on TCGA OC dataset and identified immune-related pathways [37]. In this article, GSEA analysis was performed between the high- and low-CACNA1C expression datasets to explore how CACNA1C participates in the pathogenesis of OC. Finally, five pathways that exhibited significant differential enrichment in the high-CACNA1C expression phenotype were detected, containing Base excision repair signaling pathway, Glyoxylate and dicarboxylate metabolism signaling pathway, Mismatch repair signaling pathway, Nucleotide excision repair signaling pathway and Oxidative phosphorylation signaling pathway.

In terms of genetic alteration, it had been found to be associated with the development and progress of various cancers [38–40]. Shen et al. revealed that genetic and epigenetic alterations of p33ING1b were related to the pathogenesis of OC [41]. Pongstaporn et al. found that the GSTO2 gene polymorphism might be easily associated with OC risks [42]. Our results shed light on that the genetic alteration frequency of CACNA1C was about 10 % in OC by means of the cBioPortal tool from TCGA dataset. Moreover, K-M survival analyses showed that altered CACNA1C groups were significantly associated with OS, progression-free survival, disease-free survival and disease-specific survival, compared with unaltered groups in OC. All of these indicated that genetic alterations of CACNA1C might play vital roles in OC. Furthermore, we also identified altered CACNA1C-related signaling pathways, including Cell-cycle-signaling-pathway, RTK-RAS-PI(3)K-pathway and Notch-signaling-pathway.

A growing number of studies reported that MSI, TMB and TNB played important roles in tumorigenesis and they could serve as important predictive biomarkers for the application of immunotherapy [43–45]. Fan et al. developed and verified a TMB-related prognostic signature, which could be a promising prognostic signature for OC prognosis [46]. Xiao et al. suggested that mismatch repair (MMR) deficient OC patients might be good candidates for anti-PD-1/PD-L1 therapy, characterized by increased tumor-infiltrating lymphocytes and MSI phenotype [47]. Based on OC samples from the TCGA cohort, we also explored the associations between the CACNA1C expression and MSI, TMB, TNB. Our results shed light on that the CACNA1C expression was significantly related to MSI in OC, while it had nothing to do with TMB or TNB.

As for immunity, four aspects were analyzed respectively, including tumor immune infiltration, tumor microenvironment, immune checkpoint molecules and immune cells pathway. Previous articles reported that tumor immune infiltration and tumor microenvironment might be involved in OC tumorigenesis and response to immunotherapy [48, 49]. Our results indicated that the CACNA1C expression was significantly associated with B cell infiltration, CD4 + T cell infiltration, neutrophil infiltration, macrophage infiltration, and dendritic cell infiltration for tumor immune infiltration and markedly related to ESTIMATEScore and StromalScore for tumor microenvironment. Correlations between gene expression and immune checkpoint molecules or immune cells pathway were also analyzed and various genes had been identified in OC [50, 51]. Our results indicated that the CACNA1C expression was remarkably linked to ADORA2A, CD276, CD28, NRP1, TNFRSF8, TNFSF4, Central memory CD8 T cells pathway and Type 17 T helper cells pathway.

As for its potential mechanism, our results indicated that CACNA1C could be a prognostic predictor of OS in OC and it was closely related to immunity, having an effect on oxidative phosphorylation and DNA repair activities including base excision repair, mismatch repair and nucleotide excision repair signaling pathways. As reported by previous article, oxidative phosphorylation could serve as a target for novel therapeutic strategies against OC [52]. DNA repair activities were also revealed to be the most common cause of hereditary OC [53–55]. Further experimental researches were required to verify these findings.

The strength of this article was that it was the first time for us to explore the roles of CACNA1C in OC and we further validated this gene in the ICGC dataset, qRT-PCR results and the HPA database. This study had several limitations too. Firstly, due to the absence of normal OC samples in TCGA, we had to analyze CACNA1C single gene data in combination of TCGA and GTEx datasets. This inevitably would lead to some biases. Secondly, our study was a retrospective study based on public databases without further experimental verification from our own samples, except for qRT-PCR. Last but not least, a larger sample size of patients was required to verify the prognostic roles of CACNA1C in OC and to confirm its associations with immunity.

## Conclusions

Taken together, our results shed light on that CACNA1C could be a prognostic predictor for patients with OC and five signaling pathways regulated by CACNA1C were also identified. Moreover, our analyses of CACNA1C indicated statistical correlations of CACNA1C expression with clinical prognosis, genetic alteration, MSI, tumor immune infiltration, tumor microenvironment, immune checkpoint molecules and immune cells pathway, helping to understand its role in OC from the perspective of clinical tumor samples. Further experimental validations were still required to mine the molecular mechanisms associated with CACNA1C in OC.

## Supplementary Information

Additional file 1: **Figure S1.** Altered CACNA1C-related signaling pathways; (**A**) Cell-cycle-signaling-pathway; (**B**) RTK-RAS-PI(3)K-pathway; (**C**) Notch-signaling-pathway.

## Data Availability

The RNA-sequencing data and corresponding clinical information were downloaded from The Cancer Genome Atlas (TCGA) database (https://portal.gdc.cancer.gov/) and the International Cancer Genome Consortium (ICGC) database (https://icgc.org/).
