# Supplementary figures and images for: CACNA1C is a prognostic predictor for patients with ovarian cancer
Source: J Ovarian Res. 2021 Jul 1;14:88. doi: 10.1186/s13048-021-00830-z (PMC8252246; doi:10.1186/s13048-021-00830-z)

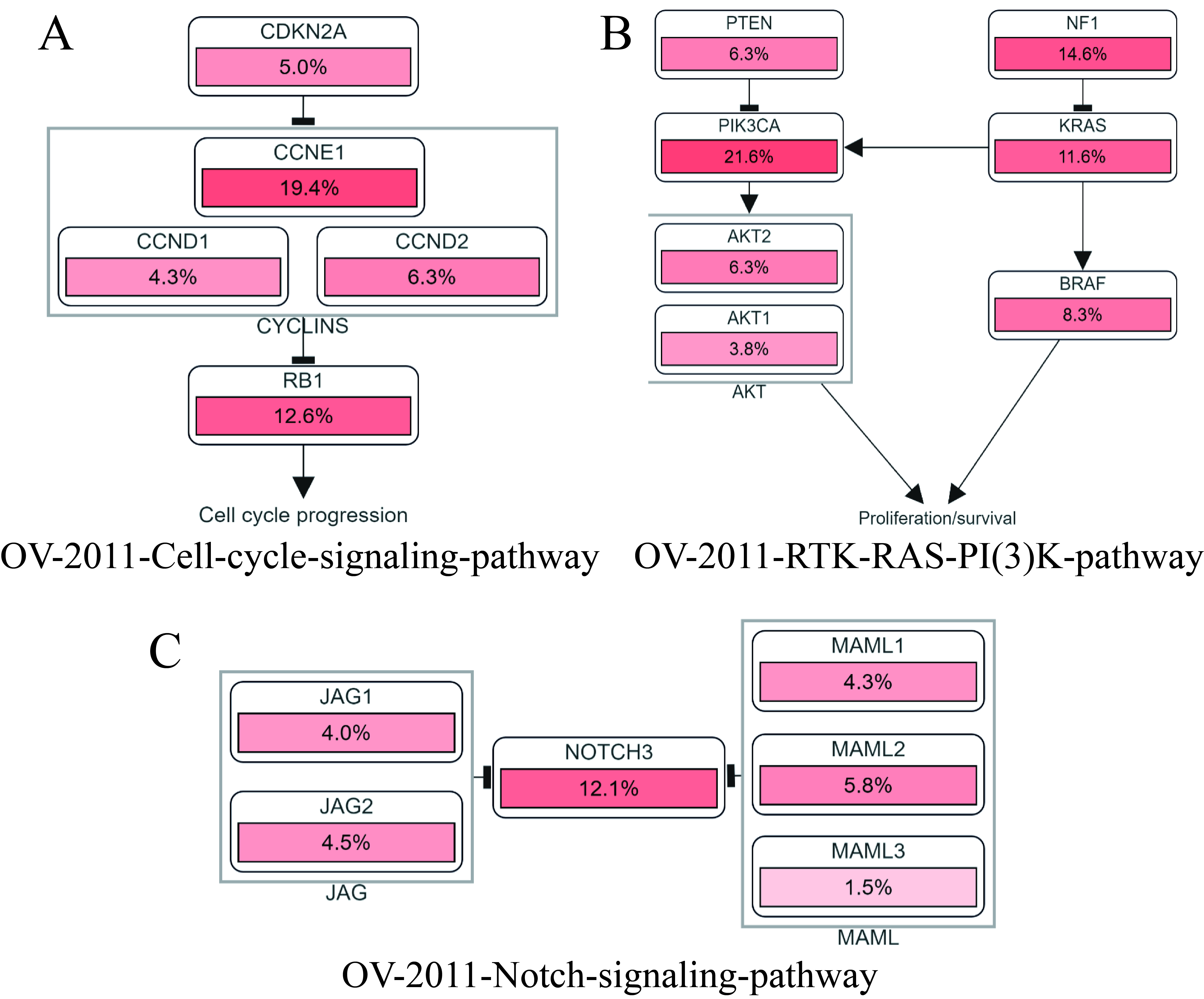

Supplement: Supplementary file 1 — Figure S1. Altered CACNA1C-related signaling pathways; (A) Cell-cycle-signaling-pathway; (B) RTK-RAS-PI(3)K-pathway; (C) Notch-signaling-pathway. [file 13048_2021_830_MOESM1_ESM.tif]
